# Supplementary material for: Development of Innate-Immune-Cell-Based Immunotherapy for Adult T-Cell Leukemia–Lymphoma
Source: Cells. 2024 Jan 10;13(2):128. doi: 10.3390/cells13020128 (PMC10814776; doi:10.3390/cells13020128)
Supplement: Supplementary file 1 [file cells-13-00128-s001.zip › cells-2758344-supplementary.pdf]

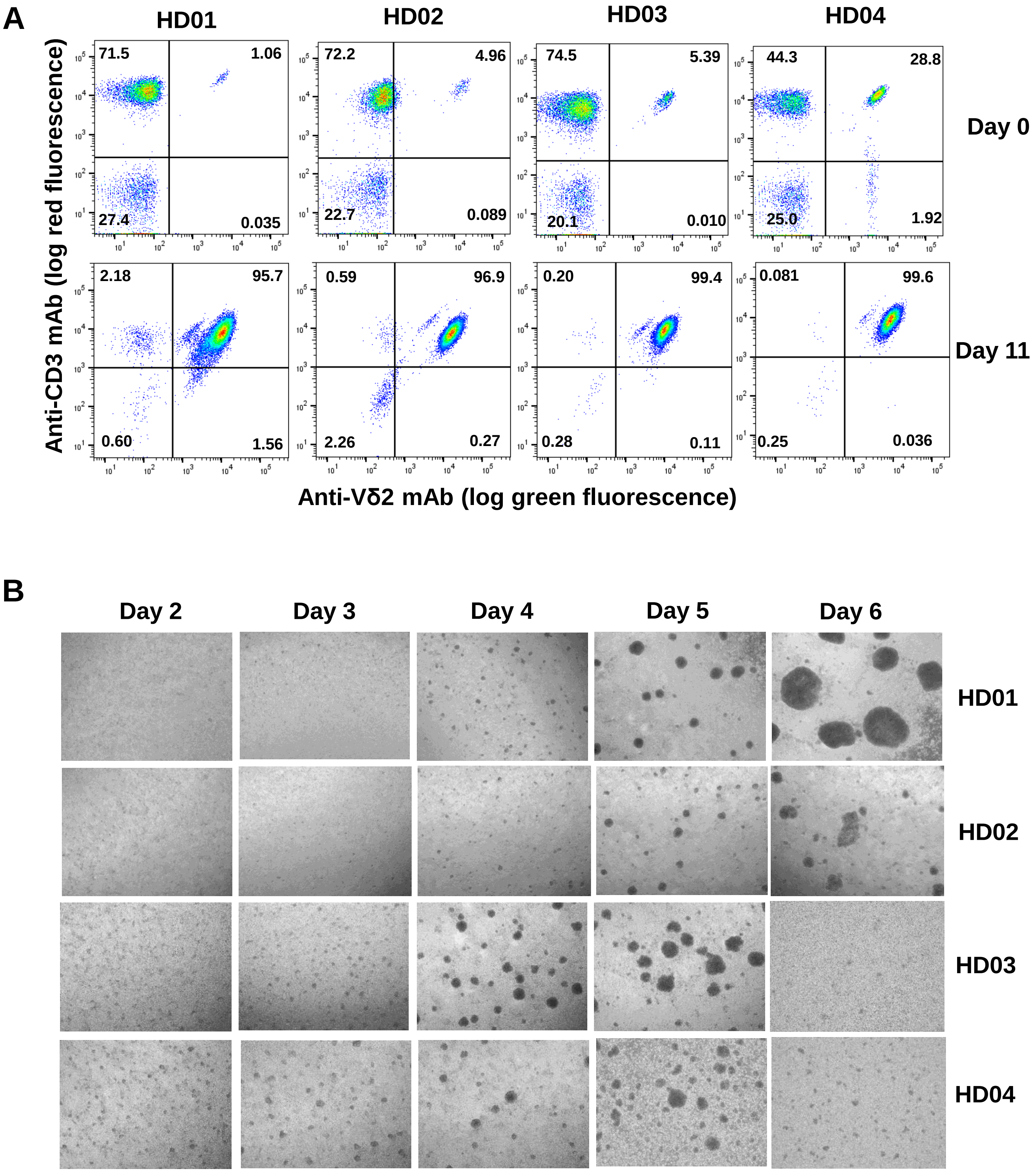

Supplementary Fig. 1. Expansion of γδ T cells from HDs with PTA/IL-2. (A) Flow cytometric analyses of PTA/IL-2-mediated expansion of γδ T cells derived from HDs. Before and after expansion for 11 days with PTA/IL-2, the cells were stained with phycoerythrin (PE)-labeled anti-CD3 mAb and fluorescein isothiocyanate (FITC)-labeled anti-Vδ2 mAb and analyzed through a FACS Lyric flow cytometer. **(B) PTA-mediated clustering of γδ T cells.** After stimulation/expansion with PTA/IL-2, cell clustering was monitored under a microscope equipped with a CCD camera.

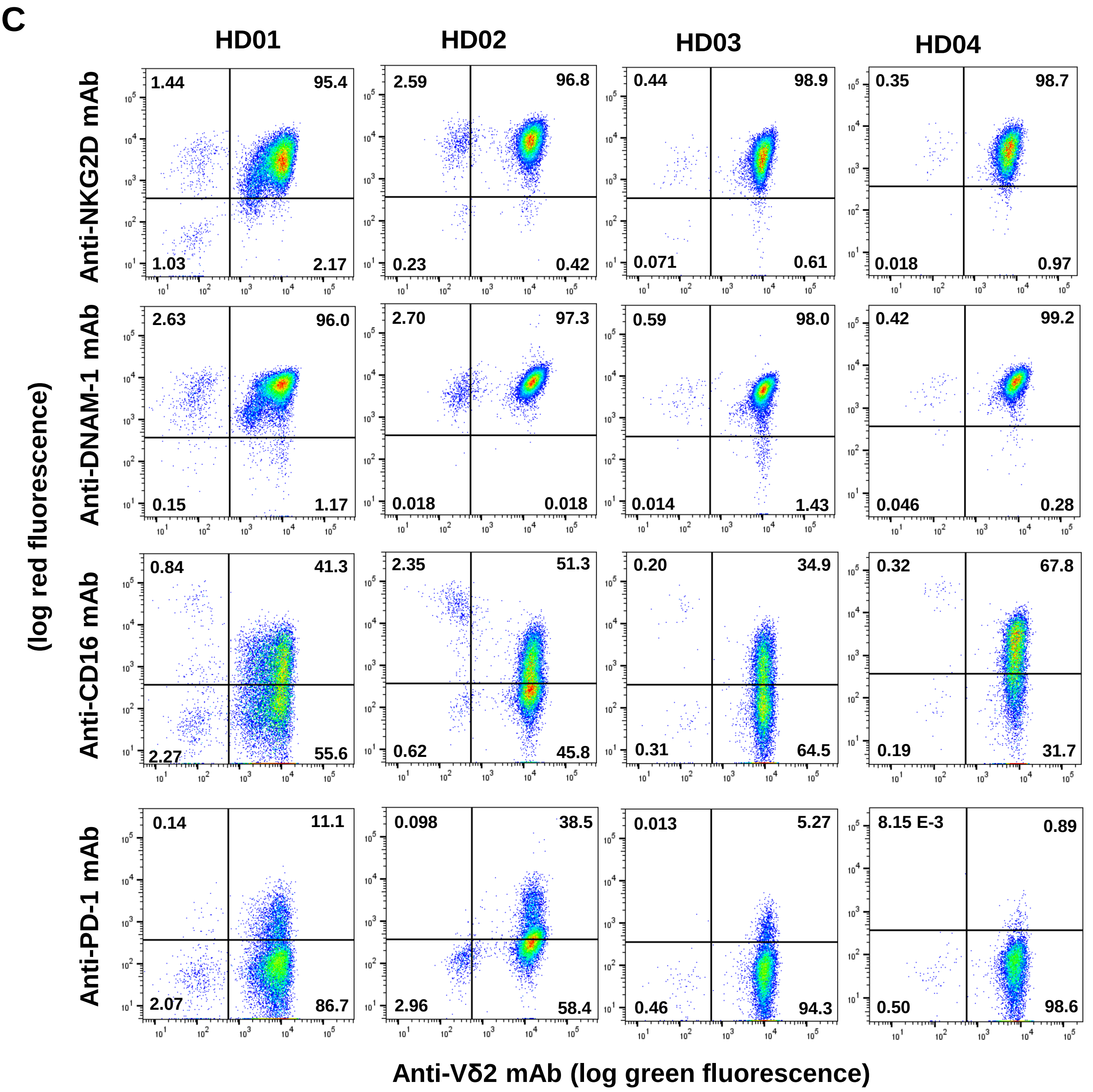

Supplementary Fig. 1. Expansion of γδ T cells from HDs with PTA/IL-2. (C) Flow cytometric analyses of cell surface markers on PTA/IL-2-expanded γδ T cells. After stimulation/expansion with PTA/IL-2 for 11 days, the cells were stained with PE-labeled anti-NKG2D, DNAM-1, CD16, or PD-1 mAb and FITC-labeled anti-Vδ2 mAb and analyzed through a FACS Lyric flow cytometer.

Materials and Methods for Supplementary Fig. 1.

(A) Preparation of PBMC: Peripheral blood samples (14 mL) were collected from HDs using heparin-containing blood collection tubes and transferred into a 15 mL conical tube, which was centrifuged at 2,200 rpm for 10 min at room temperature. After removing the plasma, the cell pellets were diluted with phosphate-buffered saline (PBS) to a volume of 20 mL. The diluted blood was loaded on 20 mL of Ficoll-Paque Plus (Cytiva, Shinjuku-ku, Tokyo, Japan) in a 50 mL conical tube and centrifuged at 1,700 rpm (600 × g) for 30 min at room temperature without acceleration and deceleration. Fluffy layers (lymphocyte fractions) were collected and diluted with 35 mL of PBS in a 50 mL conical tube, which was centrifuged at 2,200 rpm for 10 min at 4 °C. After the supernatant was discarded, the cell pellets were dispersed with tapping and resuspension in 13 mL of PBS in a 15 mL conical tube. After centrifugation at 1,700 rpm for 5 min at 4 °C, the cells were resuspended in 7.2 mL of Yssel's medium supplemented with 10% heat-inactivated human AB serum [60] to achieve a maximum cell concentration of 1×10^7 cells/mL. Of the PBMC suspension, 1.2 mL was used for the flow cytometric analysis.

(B) Derivation of γδ T cells: The PBMC suspension (6 mL) was placed in 4 wells of a 24-well plate (Corning Inc., Corning, NY), to which was added 1.5 µL each of 1 mM PTA stock solution (Techno Suzuta Co., Ltd., Heiwa-machi, Nagasaki, Japan) in dimethyl sulfoxide (DMSO) (Nacalai Tesque Inc., Nakagyo-ku, Kyoto, Japan), resulting in a final concentration of 1 µM. The cells were observed under a microscope (Nikon Corp., Minato-ku, Tokyo, Japan) every day during incubation. The plate was incubated at 37 °C with 5% CO_2 overnight, and IL-2 (Shionogi Pharmaceutical Co., Ltd., Chuo-ku, Osaka, Japan) was added to each well, to obtain a concentration of 100 U/mL, from day 1 to day 9. On day 2, the medium was replaced with fresh Yssel's medium supplemented with 10% heat-inactivated human AB serum to remove any residual PTA/DMSO that might affect the growth in γδ T cells. Whenever the cell density increased to confluency, the cell suspensions were diluted 2-fold with Yssel's medium supplemented with heat-inactivated 10% human AB serum (when culturing in wells) or complete RPMI1640 medium (when culturing in flasks) and split to new wells or flasks until day 9. The γδ T cells were then harvested on day 11. After the flow cytometric analysis, the remaining cells were resuspended in cryo-preservation media, placed at -80 °C, and stored in liquid nitrogen until used.

(C) Flow cytometric analysis: The cell suspensions, 110 µL each (ca. 1.0×10^5 cells), were dispensed into a 96-well round-bottom plate (Corning Inc.) and centrifuged at 1,700 rpm and 4 °C for 2 min. After the supernatants were removed, the cell pellets were dispersed by vortexing. The cells were stained with monoclonal antibodies (mAbs), 3 µL each, in 47 µL of PBS/2% fetal calf serum (FCS, Merk, Darmstadt, Hessen, Germany). Immunohistochemical staining was performed using fluorescein isothiocyanate (FITC)-conjugated anti-TCR Vδ2 mAb (Beckman Coulter Inc., Pasadena, CA); phycoerythrin (PE)-conjugated anti-CD3 mAb (Thermo Fisher Scientific Inc.), and anti-NKG2D, anti-DNAM-1, and anti-CD16 mAbs (BioLegend Japan, Bunkyo-ku, Tokyo, Japan), unlabeled anti-PD-1 mAb (Medical & Biological Laboratories Co., Ltd., Minato-ku, Tokyo, Japan); and R-PE-conjugated anti-mouse immunoglobulin Ab (Agilent Technologies, Santa Clara, CA). After the plate was incubated on ice for 15 min, the cells were centrifuged with 100 µL of PBS at 1,700 rpm for 2 min at 4 °C. After the supernatants were removed, the cell pellets were dispersed by vortexing. The cells were, subsequently, washed three times with 200 µL of PBS via centrifugation at 1,700 rpm for 2 min at 4 °C, and they were resuspended in 200 µL of 1% paraformaldehyde in PBS. The cell suspensions were passed through a mesh filter membrane and analyzed using a FACS Lyric flow cytometer (Becton Dickenson, Franklin, Lakes, NJ). The cell population was visualized with FlowJo ver. 10.8.1 (FlowJo LLC, Ash-land, OR).

Results for Supplementary Fig. 1. Expansion of γδ T cells from HDs with PTA/IL-2.

(A) Flow cytometric analyses of PTA/IL-2-mediated expansion of γδ T cells derived from HDs. As HDs,12 males and 4 females were enrolled in this study. The median age at the time of blood sampling was 34 years (range, 27–58 years). PBMC were isolated through a standard Ficoll density centrifugation procedure, of which 4 representative results of flow cytometric analyses are depicted in the upper panels. The proportions of Vδ2-expressing γδ T cells (termed γδ T cells hereafter) in PBMC on day 0 were 1.06%, 4.96%, 5.39%, and 28.8% for HD01–04, respectively, and the median proportion of γδ T cells in $CD3^+$ lymphocyte fractions was 3.82% (range, 0.56%–39.4%).

After the PBMCs were stimulated with PTA, a nitrogen-containing bisphosphonate prodrug, and IL-2 for 11 days, the proportions of γδ T cells increased to 95.7%, 96.9%, 99.4%, and 99.6% for HD01–04, respectively, as shown in the lower panels. The median proportion of γδ T cells in $CD3^+$ lymphocyte fractions increased to 99.19%, with a range of 94.56%-99.92%. The median number of γδ T cells (per mL of blood) before and after expansion was 6.3×10^4 (range: 6×10^3–4.75×10^5) and 1.04×10^8 (range: 4×10^6–2.03×10^8), respectively, and the median expansion rate of the γδ T cells was 1091-fold (range: 415–4835). Consistent with our previous results, a large number of highly purified γδ T cells were obtained using the PTA/IL-2 stimulation/expansion system [50] when the proportion of γδ T cells in the $CD3^+$ lymphocyte fractions was well above 1%.

(B) PTA-mediated clustering of γδ T cells. Microscopic analyses revealed that the cells started to form clusters 3 to 5 days following PTA/IL-2 stimulation.

(C) Flow cytometric analyses of cell surface markers on PTA/IL-2-expanded γδ T cells. Since γδ T cells are categorized into both innate immune cells and adaptive immune cells, we next examined the cell surface expression of NK receptors [62-67], such as natural killer group 2 member D (NKG2D, CD314), DNAX accessory molecule-1 (DNAM-1, CD226), and CD16 (FcγRIIIA), whose expressions are inexorably linked to NK cells. Based on the flow cytometric analyses, essentially all of the PTA/IL-2-expanded γδ T cells expressed NKG2D and DNAM-1, as shown in Supplementary Figure 1C. The median proportions of NKG2D and DNAM-1 in the γδ T cells were 98.93% (range: 96.04%–99.96%) and 98.97% (range: 96.5%–99.99%), respectively. On the contrary, the majority of γδ T cells failed to express a high level of FasL (CD95L) and TRAIL (human TNF-related apoptosis-inducing ligand) (data not shown). The median proportions of FasL and TRAIL in γδ T cells were 0.09% (range: 0.03%–4.78%) and 0.19% (range: 0.03%–5.27%), respectively. γδ T cells expressed CD16 to different degrees depending on the individual, and the median proportion of CD16-expressing γδ T cells was 38.9% (range: 7.5%–70.9%). It has been reported that programmed death-1 $(PD\text{-}1)^+$ γδ T cells could produce a significantly higher level of IL-2 in response to (*E*)-4-hydroxy-3-methylbut-2-enyl diphosphate (HMBPP) than $PD\text{-}1^-$ γδ T cells did, and the expression of PD-1 on γδ T cells generally attained the maximum within 3 days after stimulation with pyrophosphomonoester antigens and gradually declined thereafter [68-69]. We, thus, examined the level of PD-1 expression in γδ T cells after stimulation/expansion with PTA/IL-2. It is of note that only a small portion of γδ T cells expressed a low level of PD-1, and the median proportion of PD-1 in γδ T cells was 11.35% (range: 0.9%–59.4%).

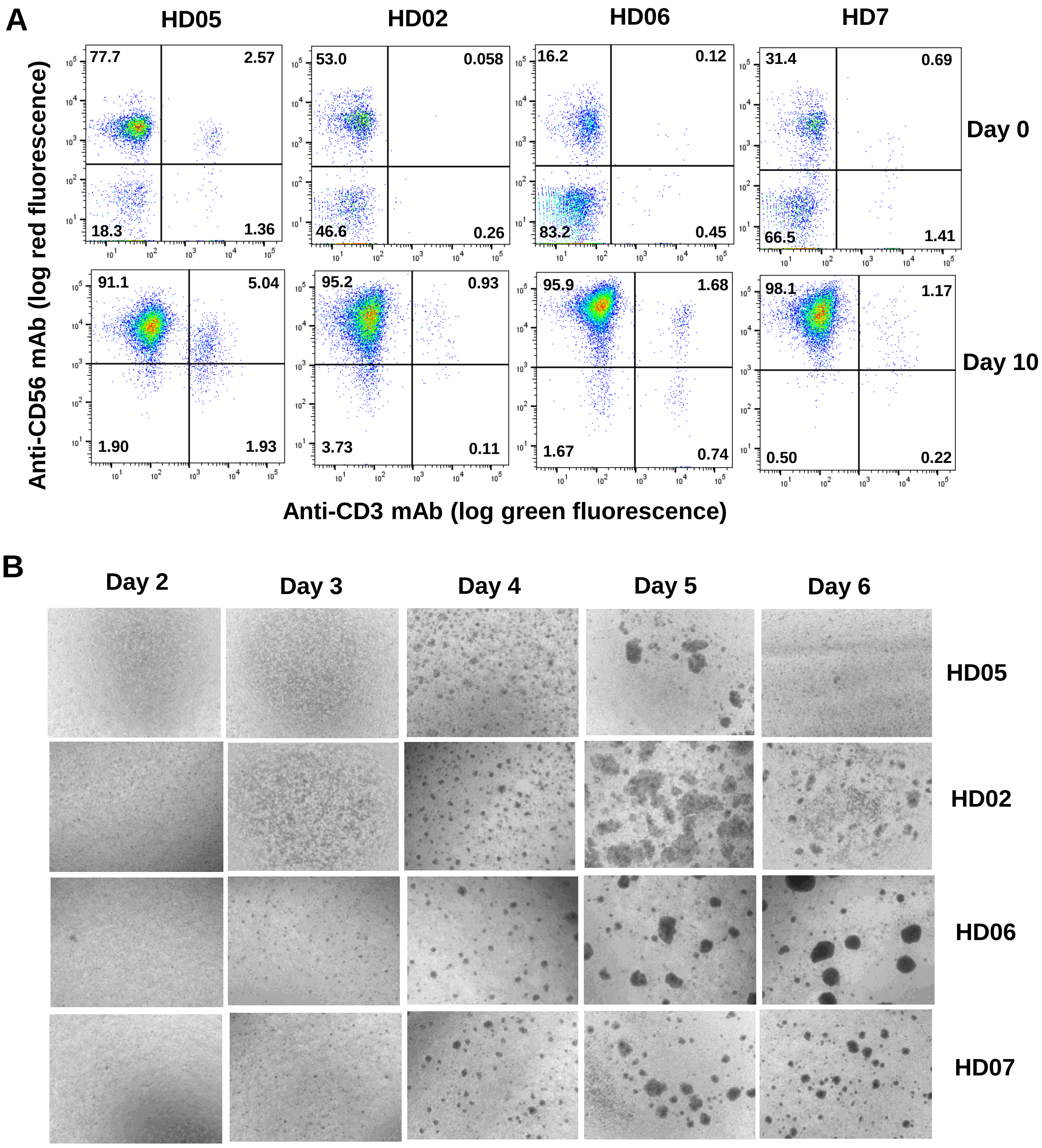

Supplementary Fig. 2. Expansion of NK cells from HDs with IL-2/IL-18. (A) Flow cytometric analyses of IL-2IL-18-mediated expansion of NK cells derived from HDs. Before and after expansion for 10 days with IL-2/IL-18, the cells were stained with PE-labeled anti-CD56 mAb and FITC-labeled anti-CD3 mAb and analyzed through a FACS Lyric flow cytometer. **(B) IL-2/IL-18-mediated clustering of NK cells.** After stimulation with IL-2/Il-18, the cell clustering was monitored under a microscope equipped with a CCD camera.

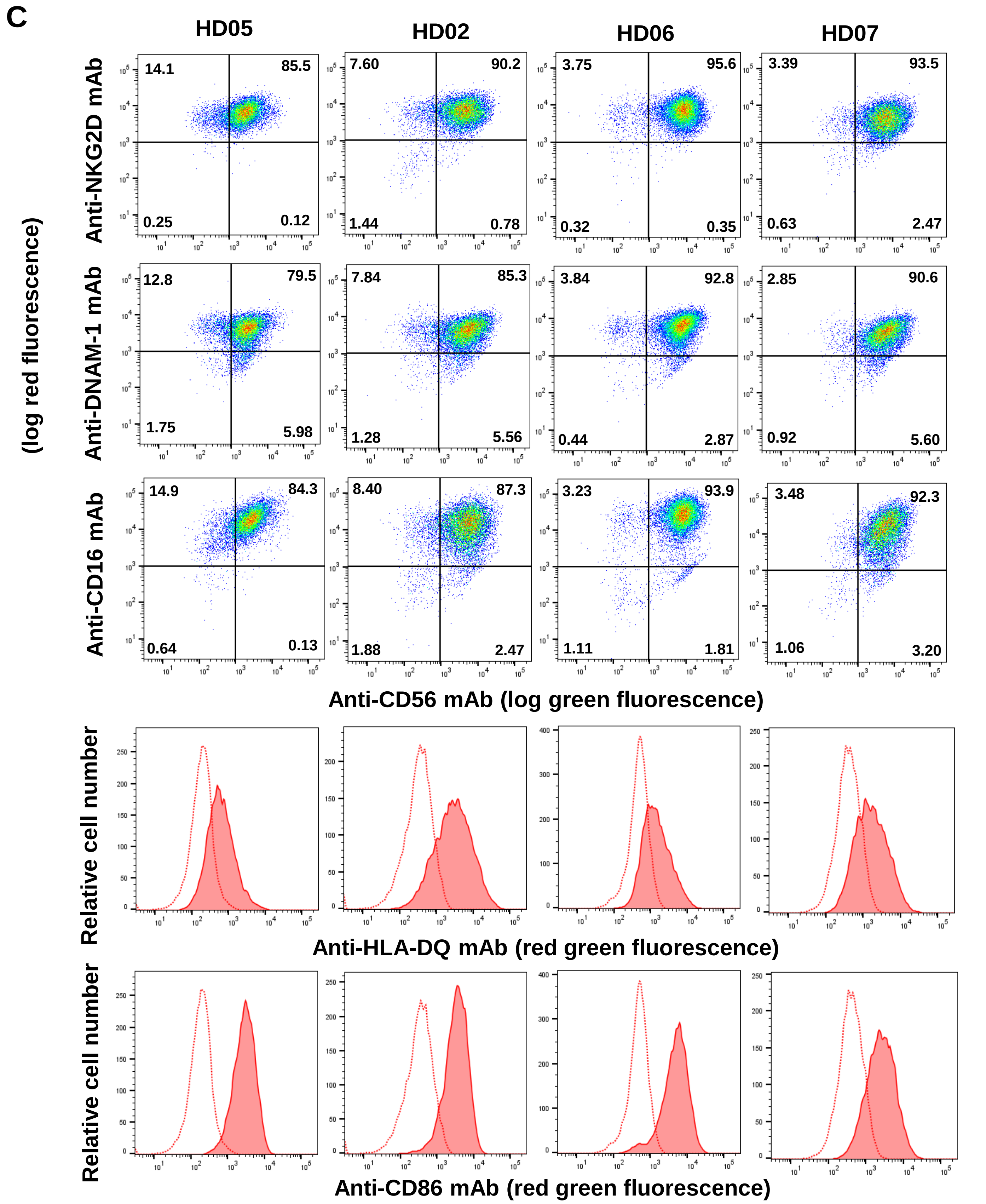

Supplementary Fig. 2. Expansion of NK cells from HDs with IL-2/IL-18. (C) Flow cytometric analyses of cell surface markers on IL-2/IL-18-expanded NK cells. After expansion with IL-2/IL-18 for 10 days, the cells were stained with PE-labeled anti-NKG2D, DNAM-1, CD16, HLA-DQ, or CD86 mAb and FITC-labeled anti-CD56 mAb and analyzed through a FACS Lyric flow cytometer.

Materials and Methods for Supplementary Fig. 2.

(A) Derivation of NK cells: PBMCs were prepared as described in Supplementary Fig. 1. After 6 mL of the PBMC suspension, in a 15 mL conical tube, was centrifuged at 1,700 rpm at 4 ^{o}C for 5 min, the supernatant was removed and the cell pellets were dispersed by tapping and resus-pended in 200 μL of PBS/0.5% BSA/2 mM EDTA. To the PBMC suspension was added 100 μL of anti-CD3 MACSBeads (Miltenyi Biotech, Bergisch-Gladbach, Germany) and the tube was placed at 4 ^{o}C. After 15 min, 10 mL of PBS/0.5% BSA/2 mM EDTA was added to the cell suspension. The tube was then centrifuged at 300 × g for 10 min at 4 ^{o}C and the supernatant was removed. The cell pellets and beads were dispersed by tapping and resuspended in 1 mL of PBS/0.5% BSA/2 mM EDTA. The cell/bead suspension was loaded onto an LD Column (Miltenyi Biotech), which had been attached to a magnet holder and equilibrated with 2 mL of PBS/0.5% BSA/2 mM EDTA. The $CD3^-$ cells were eluted with 2 × 1 mL of PBS/0.5% BSA/2 mM EDTA into a 15 mL conical tube, to which was added 6 mL of Yssel's medium supplemented with 10% human AB serum. After the tube was centrifuged at 1,700 rpm for 5 min at 4 ^{o}C, the $CD3^-$ cells were resuspended in 5 mL of Yssel's medium supplemented with 10% heat-inactivated human AB serum. Of the cell suspension, 1.5 mL was used for the flow cytometric analysis. The rest of the cell suspension was centrifuged at 1,700 rpm for 5 min at 4 ^{o}C. After the supernatant was removed, the cell pellets were dispersed by tapping and resuspended in 3 mL of Yssel's medium supplemented with 10% heat-inactivated human AB serum. The PBMC suspension (3 mL) was placed in 2 wells of a 24-well plate, to which was added 100 IU/mL IL-2 and 100 ng/mL recombinant human IL-18 (Techno Suzuta Co., Ltd), expressed in E. coli from day 0 to day 8. Cell passages were conducted based on cell confluency until day 8. Then, the NK cells were harvested on day 10 and analyzed using flow cytometry. The NK cells were placed at -80 ^{o}C and then stored in a liquid nitrogen tank until used.

(B) Flow cytometric analysis: Immunohistochemical staining was performed using FITC-conjugated anti-CD3 mAb (Thermo Fisher Scientific Inc.) and anti-CD56 mAb (BioLegend, San Diego, CA); and phycoerythrin (PE)-conjugated anti-CD56, anti-NKG2D, anti-DNAM-1, anti-CD16, anti-HLA-DQ, and anti-CD86 mAbs (BioLegend). The stained cells were analyzed using a FACS Lyric flow cytometer (Becton Dickenson) and the cell population was visualized with FlowJo ver. 10.8.1 (FlowJo LLC) as described in Supplementary Fig. 1.

Results for Supplementary Fig. 2. Expansion of NK cells from HDs with IL-2/IL-18.
(A) Flow cytometric analyses of IL-2IL-18-mediated expansion of NK cells derived from HDs. PBMC were prepared from 10 HDs as described in Supplementary Fig. 1, then $CD3^+$ cells were depleted using anti-CD3 mAb-coated beads. Four representative results of flow cytometric analyses of the $CD3^-$ depleted PBMCs (HD02, 05–07) are shown in the upper panels. The proportions of $CD3^-CD56^+$ NK cells after treatment with anti-CD3 mAb beads were 77.7%, 53.0%, 16.2%, and 31.4%, respectively, and the median proportion of NK cell fractions after CD3 removal was 49.9% (range: 31.4% – 92.3%).

After the $CD3^-$ PBMCs were stimulated/expanded with IL-2 and IL-18 for 10 days. The proportion of NK cells increased to 91.1%, 95.2%, 95.9%, and 98.1%, for HD02, 05–07, respectively as depicted in the lower panels. The median proportion of NK cells after expansion was 95.45%, with a range of 71.4%–98.4%. The median number of NK cells (per mL of blood) before and after expansion was 2.04×10^5 (range: 4.5×10^4–9.84×10^5) and 5×10^6 (range: 4×10^5–1.6×10^7), respectively. The median expansion rate of the NK cells was 40-fold (range: 1.1–74.5). We, thus, obtained highly purified NK cells using the IL-2/IL-18 expansion system. The expansion rate of the NK cells was, however, significantly low compared to that of γδ T cells.

(B) IL-2/IL-18-mediated clustering of NK cells. Microscopic analyses revealed that cells started to form clusters 4 to 5 days after stimulation with IL-2/IL-18.

(C) Flow cytometric analyses of cell surface markers on IL-2/IL-18-expanded NK cells. Based on flow cytometric analyses of the cell surface markers after IL-2/IL-18 stimulation, essentially all of the expanded NK cells expressed high levels of NKG2D, DNAM-1, and CD16, as shown in the upper bivariate histograms. The median proportions of NKG2D and DNAM-1 in the NK cells were 99.65% (range: 94.98%–99.86%) and 94.03% (range: 77.4%–99.04%), respectively. It is noteworthy that essentially all NK cells expressed a high level of CD16, which is in contrast to γδ T cells; in fact, the median proportion of $CD16^+$ NK cells was 96.76% (range: 59.48%–99.85%).

It was previously reported that NK cells express cell surface molecules, typically expressed on antigen-presenting cells, in response to IL-2/IL-18. In addition, the cytolytic functions of NK cells are intricately controlled by activating and inhibiting receptors, including human leukocyte antigen (HLA) molecules [70] and cluster of differentiation 86 (CD86, B7-2) [71]. We, thus, examined the expressions of HLA-DQ and CD86 on the NK cells after stimulation/expansion with IL-2/IL-18. As shown in the lower univariate histograms, more than half of the NK cells expressed high levels of HLA-DQ and CD86; the median proportions of $HLA\text{-}DQ^+$ and $CD86^+$ cells in the IL-2/IL-18-stimulated NK cells was 63.78% (range: 27.89%–93.91%) and 92.25% (range: 75.72%–93.94%), respectively.

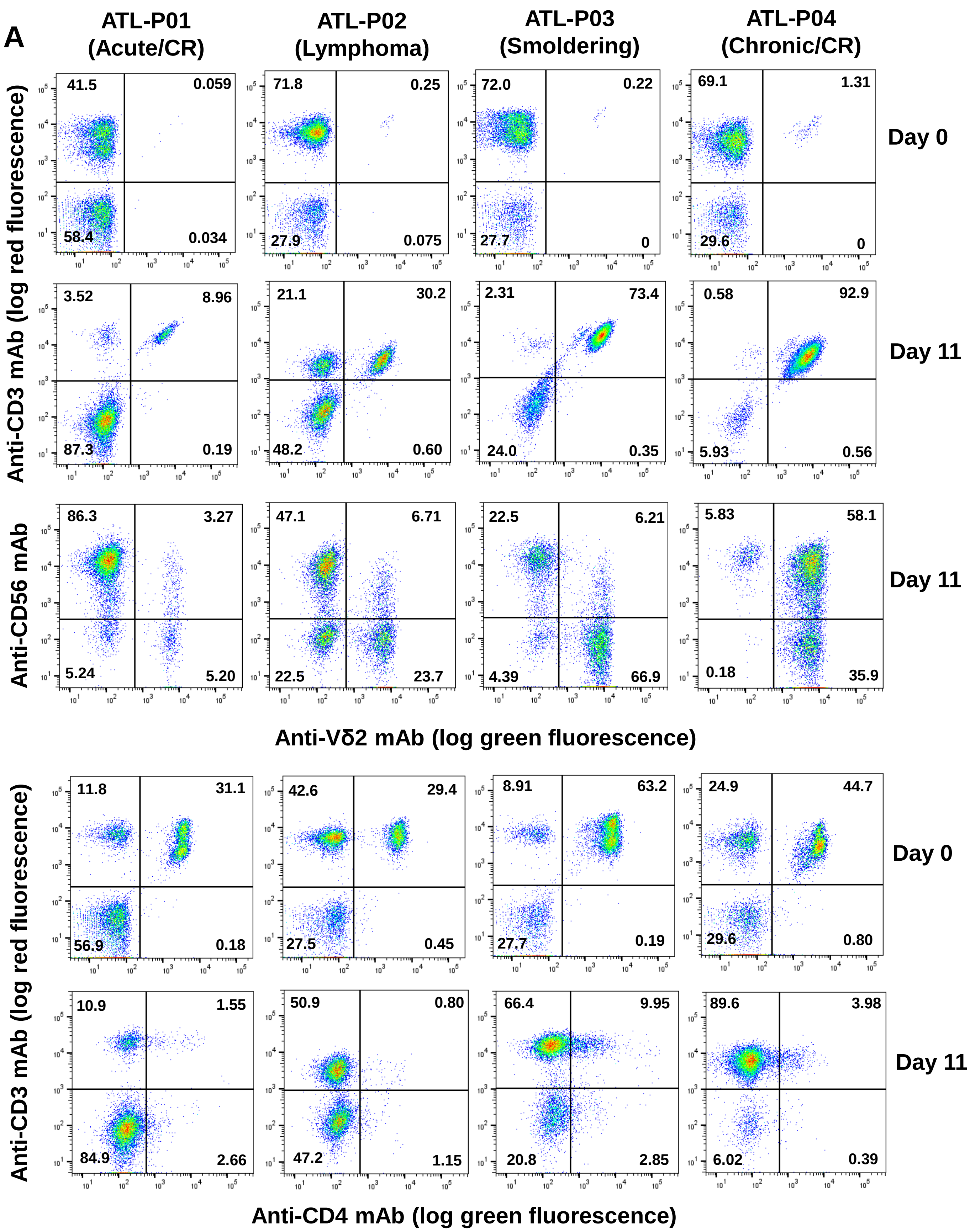
A
ATL-P01 (Acute/CR)
ATL-P02 (Lymphoma)
ATL-P03 (Smoldering)
ATL-P04 (Chronic/CR)
Day 0
Day 11
Day 11
Day 0
Day 11
Anti-CD3 mAb (log red fluorescence)
Anti-CD56 mAb
Anti-Vδ2 mAb (log green fluorescence)
Anti-CD3 mAb (log red fluorescence)
Anti-CD4 mAb (log green fluorescence)
41.5
0.059
58.4
0.034
71.8
0.25
27.9
0.075
72.0
0.22
27.7
0
69.1
1.31
29.6
0
3.52
8.96
87.3
0.19
21.1
30.2
48.2
0.60
2.31
73.4
24.0
0.35
0.58
92.9
5.93
0.56
86.3
3.27
5.24
5.20
47.1
6.71
22.5
23.7
22.5
6.21
4.39
66.9
5.83
58.1
0.18
35.9
11.8
31.1
56.9
0.18
42.6
29.4
27.5
0.45
8.91
63.2
27.7
0.19
24.9
44.7
29.6
0.80
10.9
1.55
84.9
2.66
50.9
0.80
47.2
1.15
66.4
9.95
20.8
2.85
89.6
3.98
6.02
0.39

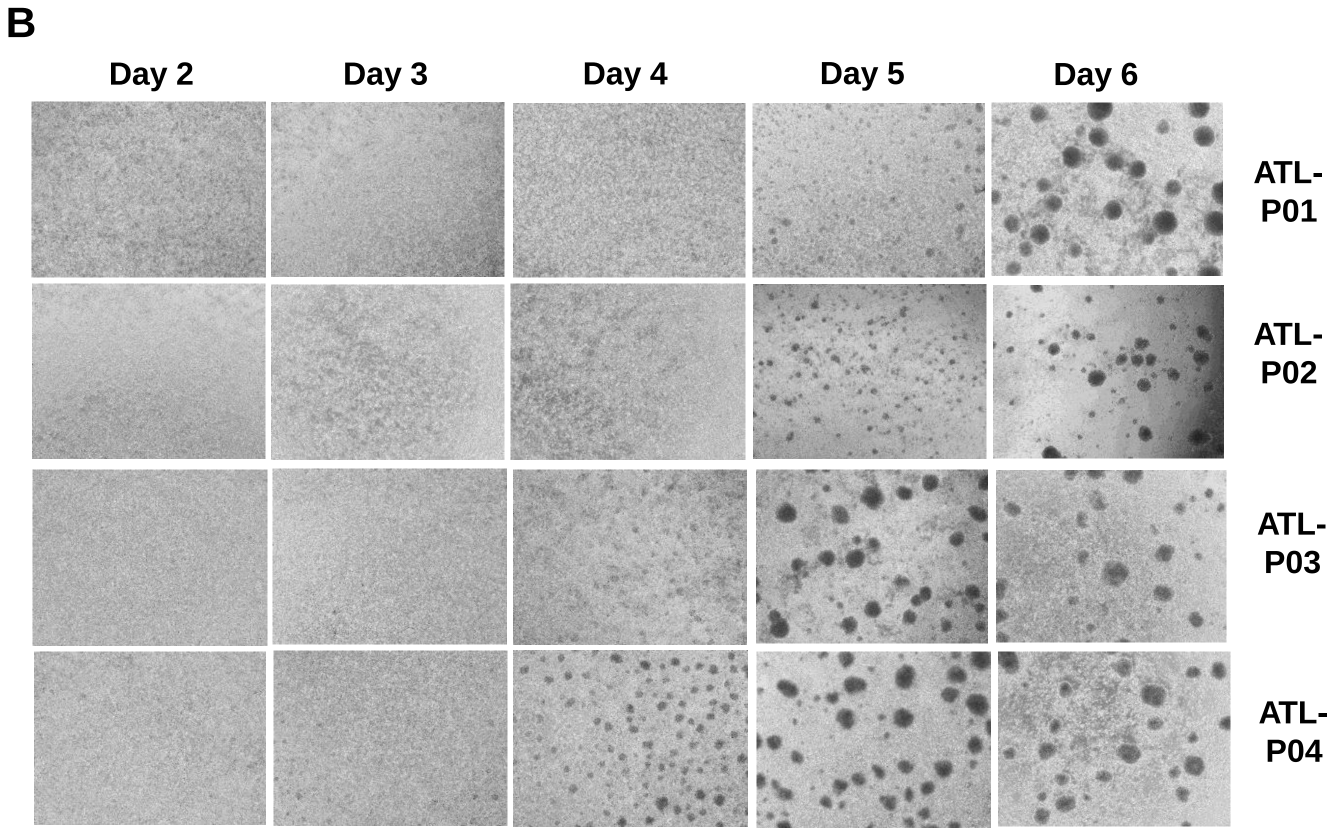

Supplementary Fig. 3. Expansion with PTA/IL-2/Il-18 of γδ T cells and NK cells derived from ATL patients. (A) Flow cytometric analyses of PTA/IL-2/IL-18-mediated expansion of γδ T cells and NK cells derived from ATL patients. PBMCs were purified from peripheral blood derived from ATL patients and stimulated/expanded with PTA/IL-2/IL-18, which were analyzed through flow cytometry. **(B) PTA/IL-2/IL-18-mediated clustering of γδ and NK cells.** After stimulation with PTA/IL-2/IL-18, the cell clustering was monitored under a microscope equipped with a CCD camera.

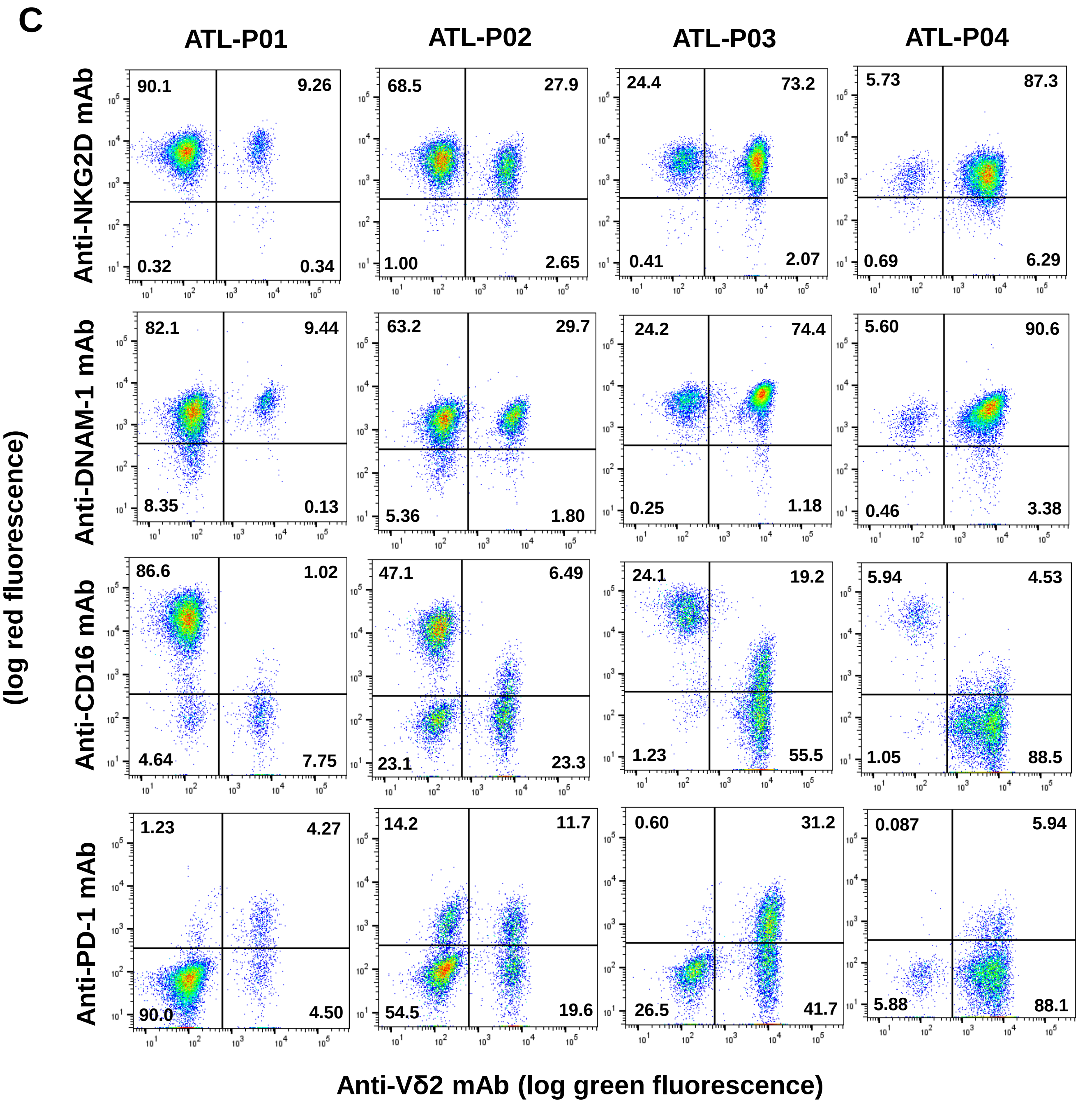

Supplementary Fig. 3. Expansion with PTA/IL-2/Il-18 of γδ T cells and NK cells derived from ATL patients. (C) Flow cytometric analyses of PTA/IL-2/IL-18-mediated expansion of γδ T cells and NK cells derived from ATL patients. After stimulation/expansion of PBMCs derived from ATL patients with PTA/IL-2/IL-18, the cells were stained with PE-conjugated anti-NKG2D, anti-DNAM-1, or anti-CD16 mAb and FITC-conjugated anti-Vδ2 mAb, or anti-PD-1 plus RPE-conjugated anti-mouse Ig ab plus FITC-conjugated anti-Vδ2 mAb and analyzed through a FACS Lyric flow cytometer.

Materials and Methods for Supplementary Fig. 3.

(A) Derivation of γδ T cells and NK cells from PBMC obtained from ATL patients: PBMC were purified from ATL patients-derived PBMC as described in Supplementary Fig. 1, from which γδ T cells and NK cells were prepared as described in Supplementary Figs. 1 and 2.

(B) Flow cytometric analysis: Immunohistochemical staining was performed using FITC-conjugated anti-TCR Vδ2 mAb (Beckman Coulter Inc.) and anti-CD4 mAbs (FUJIFILM Wako Pure Chemical Corp., Chuo-ku, Osaka, Japan); PE-conjugated anti-CD3 mAb (Thermo Fisher Scientific Inc.), and anti-CD56, anti-NKG2D, anti-DNAM-1, and anti-CD16 mAbs (BioLegend), unlabeled anti-PD-1 mAb (Medical & Biological Laboratories Co., Ltd); and R-PE-conjugated anti-mouse immunoglobulin Ab (Agilent Technologies). The stained cells were analyzed using a FACS Lyric flow cytometer (Becton Dickenson) and the cell population was visualized with FlowJo ver. 10.8.1 (FlowJo LLC) as described in Supplementary Fig. 1.

Results for Supplementary Fig. 3. Expansion with PTA/IL-2/Il-18 of γδ T cells and NK cells derived from ATL patients.

(A) Flow cytometric analyses of PTA/IL-2/IL-18-mediated expansion of γδ T cells and NK cells derived from ATL patients. : PBMC derived from 55 ATL patients (initial 25 patients plus additional 30 patients) were stimulated/expanded with PTA/IL-2/IL-18, of which 4 representative flow cytometry diagrams (ATL-P01–04) are depicted in the upper panels. The median proportion of γδ T cells in $CD3^+$ lymphocyte fractions before expansion was 0.29% (range: 0.0%–7.41%). After stimulation/expansion with PTA/IL-2/IL-18 for 11 days, the median proportion of γδ T cells in $CD3^+$ lymphocyte fractions increased to 87.99% (range: 0.55%–99.38%). The median number of γδ T cells (per mL of blood) before and after expansion was 4.2×10^3 (range: 0.0–6.6×10^4) and 3.5×10^6 (range: 2×10^3–1.75×10^8), respectively. The median expansion rate of the γδ T cells was 1998-fold (range: 4–32844). It is intriguing that $CD3^-CD56^+$ cells (corresponding to NK cells) were increased when the proportion of γδ T cells were low on day 11.

Since ATL is a mature peripheral $CD3^+CD4^+$ T-cell malignancy, we examined the proportion of $CD4^+$ T cells in $CD3^+$ lymphocyte fractions. Four representative flow cytometry diagrams are shown in the lower panels. The median proportions of $CD4^+$ T cells in $CD3^+$ lymphocyte fractions before and after expansion with PTA/IL-2/IL-18 were 72.49% (range: 30.33%–99.5%) and 6.67% (range: 0.76%–99.76%), respectively. The proportion of $CD4^+$ T cells in $CD3^+$ lymphocyte fractions was greatly reduced in most cases after the expansion with for 11 days, suggesting that a combination of γδ T cells and NK cells exhibited cellular cytotoxicity against HTLV-1-infected $CD4^+$ T cells.

(B) PTA/IL-2/IL-18-mediated clustering of γδ and NK cells. : A microscopic analysis revealed that the cells started to form clusters 3 to 6 days after stimulation.

(C) Flow cytometric analyses of PTA/IL-2/IL-18-mediated expansion of γδ T cells and NK cells derived from ATL patients. After expansion with PTA/IL-2/IL-18, essentially all the expanded γδ T cells expressed NKG2D and DNAM-1. The median proportions of NKG2D and DNAM-1 in the γδ T cells were 97.40% (range: 82.81% – 99.9%) and 98.79% (range: 92.86%–99.91%), respectively. The γδ T cells expressed CD16 to different degrees; in fact, the median proportion of CD16 in the γδ T cells was 17.7% (range: 0.98%–89.94%). In addition, the γδ T cells expressed, to different degrees, a low level of PD-1, and the median proportion of PD-1 in the γδ T cells was 29.87% (range: 2.76%–57.93%).

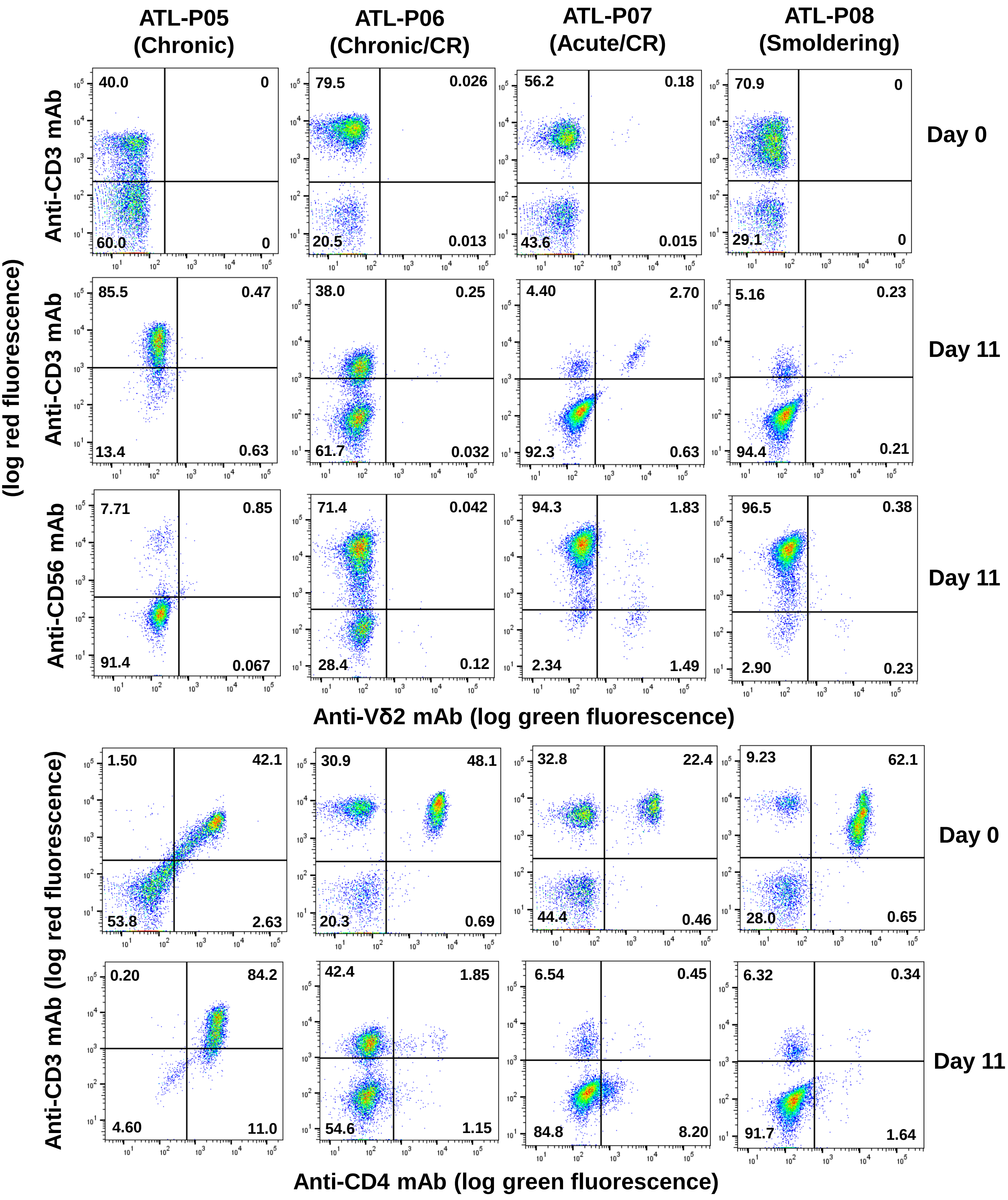

Supplementary Fig. 4. Effect of PTA/IL-2/Il-18 on the expansion of γδ T cells derived from ATL patients. Flow cytometric analyses of PTA/IL-2/IL-18-mediated expansion of γδ T cells and NK cells derived from ATL patients. PBMCs were purified from peripheral blood derived from ATL patients and stimulated/expanded with PTA/IL-2/IL-18, which were analyzed through flow cytometry.

Materials and Methods for Supplementary Fig. 4.

(A) Derivation of γδ T cells from PBMC obtained from ATL patients: PBMC were purified from ATL patients-derived PBMC as described in Supplementary Fig. 1. The PBMC suspension (6 mL) was placed in 4 wells of a 24-well plate (Corning Inc., Corning, NY), to which was added 1.5 µL each of 1 mM PTA stock solution (Techno Suzuta Co., Ltd.) in DMSO (Nacalai Tesque) to give a final concentration of 1 µM and IL-18 (Techno Suzuta Co., Ltd.) at a final concentration of 100 ng/mL. The cells were observed under a microscope (Nikon Corp., Minato-ku, Tokyo, Japan) every day during incubation. The plate was incubated at 37 ^{o}C with 5% CO_2 overnight, and IL-2 (Shionogi Pharmaceutical Co., Ltd.) and IL-18 (Techno Suzuta Co., Ltd.) were added to each well, to obtain a concentration of 100 U/mL and 100 ng/mL, respectively, from day 1 to day 9. On day 2, the medium was replaced with fresh Yssel's medium supplemented with 10% heat-inactivated human AB serum to remove any residual PTA/DMSO that might affect the growth in γδ T cells. Whenever the cell density increased to confluency, the cell suspensions were diluted 2-fold with Yssel's medium supplemented with heat-inactivated 10% human AB serum (when culturing in wells) or complete RPMI1640 medium (when culturing in flasks) and split to new wells or flasks until day 9. The γδ T cells were then harvested on day 11. After the flow cytometric analysis, the remaining cells were resuspended in cryo-preservation media, placed at -80 ^{o}C, and stored in liquid nitrogen until used.

(B) Flow cytometric analysis: Immuno-histochemical staining was performed using FITC-conjugated anti-TCR Vδ2 mAb (Beckman Coulter Inc.) and anti-CD4 mAb (FUJIFILM Wako Pure Chemical Corp.), and PE-conjugated anti-CD3 mAb (Thermo Fisher Scientific Inc.) and anti-CD56 mAb (BioLegend) and analyzed using a FACS Lyric flow cytometer (Becton Dickenson). The stained cells were analyzed using a FACS Lyric flow cytometer (Becton Dickenson) and the cell population was visualized with FlowJo ver. 10.8.1 (FlowJo LLC) as described in Supplementary Fig. 1.

Results for Supplementary Fig. 4. Effect of PTA/IL-2/Il-18 on the expansion of γδ T cells derived from ATL patients. Flow cytometric analyses of PTA/IL-2/IL-18-mediated expansion of γδ T cells and NK cells derived from ATL patients. In some ATL patients, the proportions of γδ T cells were extremely low before expansion. Flow cytometric diagrams of 4 representative ATL patients (ATL-P05–08), with extremely low proportions of γδ T cells are shown, in which γδ T cells occupied only 0%–0.18% of lymphocyte fractions. When the PBMCs were stimulated with PTA/IL-2/IL-18, only a marginal level of γδ T cell expansion was observed. Instead, in three out of four patients (ATL-P06–08), the $CD56^{+}CD3^{-}$ populations (corresponding to NK cells) increased. In the case of ATL-P05, both γδ T cells and NK cells failed to proliferate well in response to PTA/IL-2/IL-18. Among the 55 ATL patients, 3 did not respond at all to PTA/IL-2/IL-18, in which the populations of γδ T cells in $CD3^{+}$ T cells from their peripheral blood were less than 0.1% without exception. In fact, the initial frequency of γδ T cells in the $CD3^{+}$ lymphocyte fractions was less than 0.1% in 16 of 54 ATL patients (one particular case with $CD3^{-}$ HTLV-1-infected cells was excluded), whereas such a low frequency of γδ T cells was not observed in HDs. Conversely, the initial frequencies of γδ T cells in $CD3^{+}$ lymphocyte fractions were more than 1% in 12 of 54 ATL patients, whereas such conditions were met in 14 out of 16 HDs. In addition, the proportion of $CD3^{dim}CD4^{+}$ T cells in lymphocyte fractions was greatly reduced in most cases after the expansion with PTA/IL-2/IL-18 for 11 days, suggesting that a combination of γδ T cells and NK cells exhibited cellular cytotoxicity against HTLV-1-infected $CD3^{dim}CD4^{+}$ T cells.

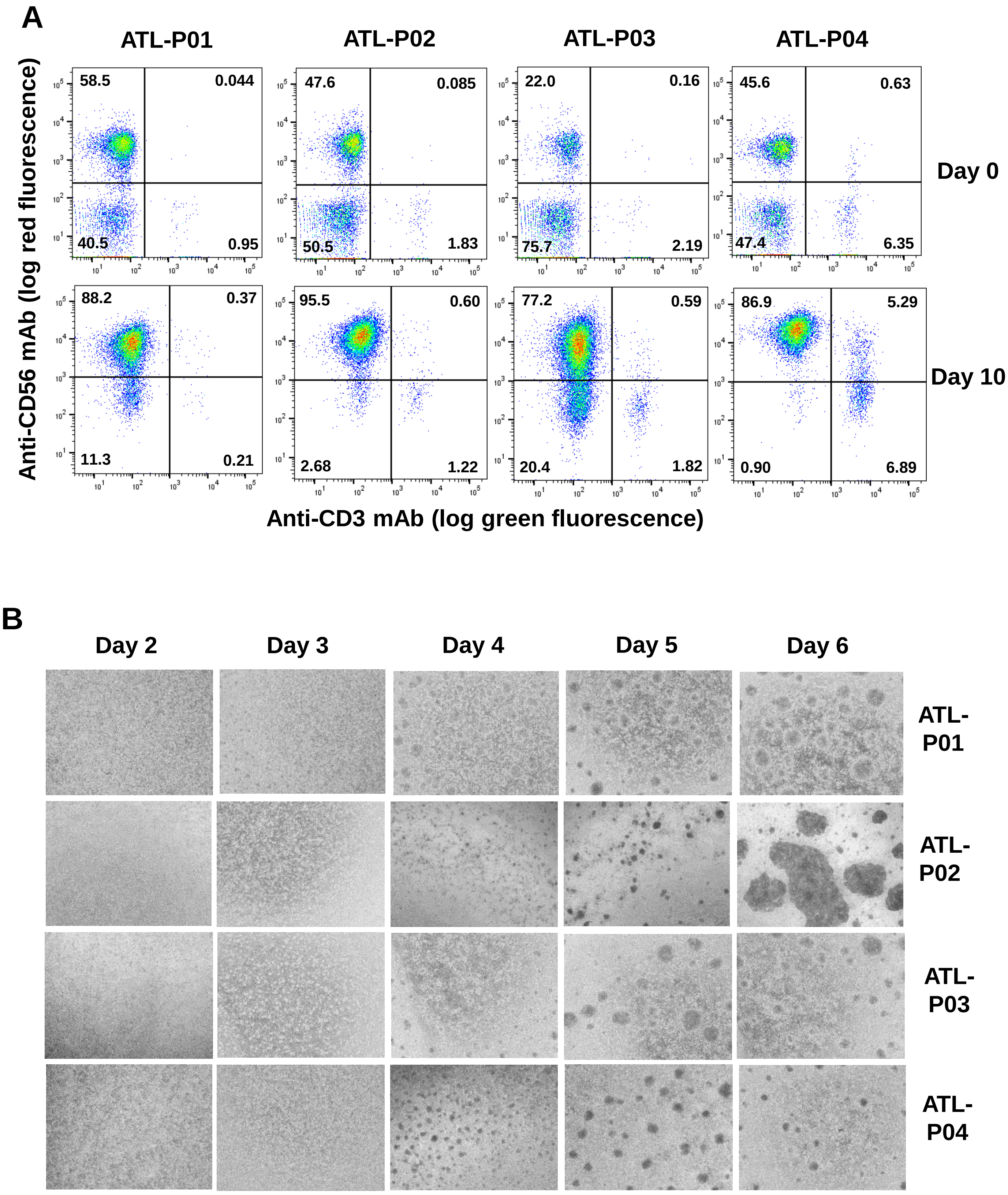
A
ATL-P01
ATL-P02
ATL-P03
ATL-P04
Day 0
Day 10
58.5
0.044
40.5
0.95
47.6
0.085
50.5
1.83
22.0
0.16
75.7
2.19
45.6
0.63
47.4
6.35
88.2
0.37
11.3
0.21
95.5
0.60
2.68
1.22
77.2
0.59
20.4
1.82
86.9
5.29
0.90
6.89
Anti-CD56 mAb (log red fluorescence)
Anti-CD3 mAb (log green fluorescence)
B
Day 2
Day 3
Day 4
Day 5
Day 6
ATL-P01
ATL-P02
ATL-P03
ATL-P04

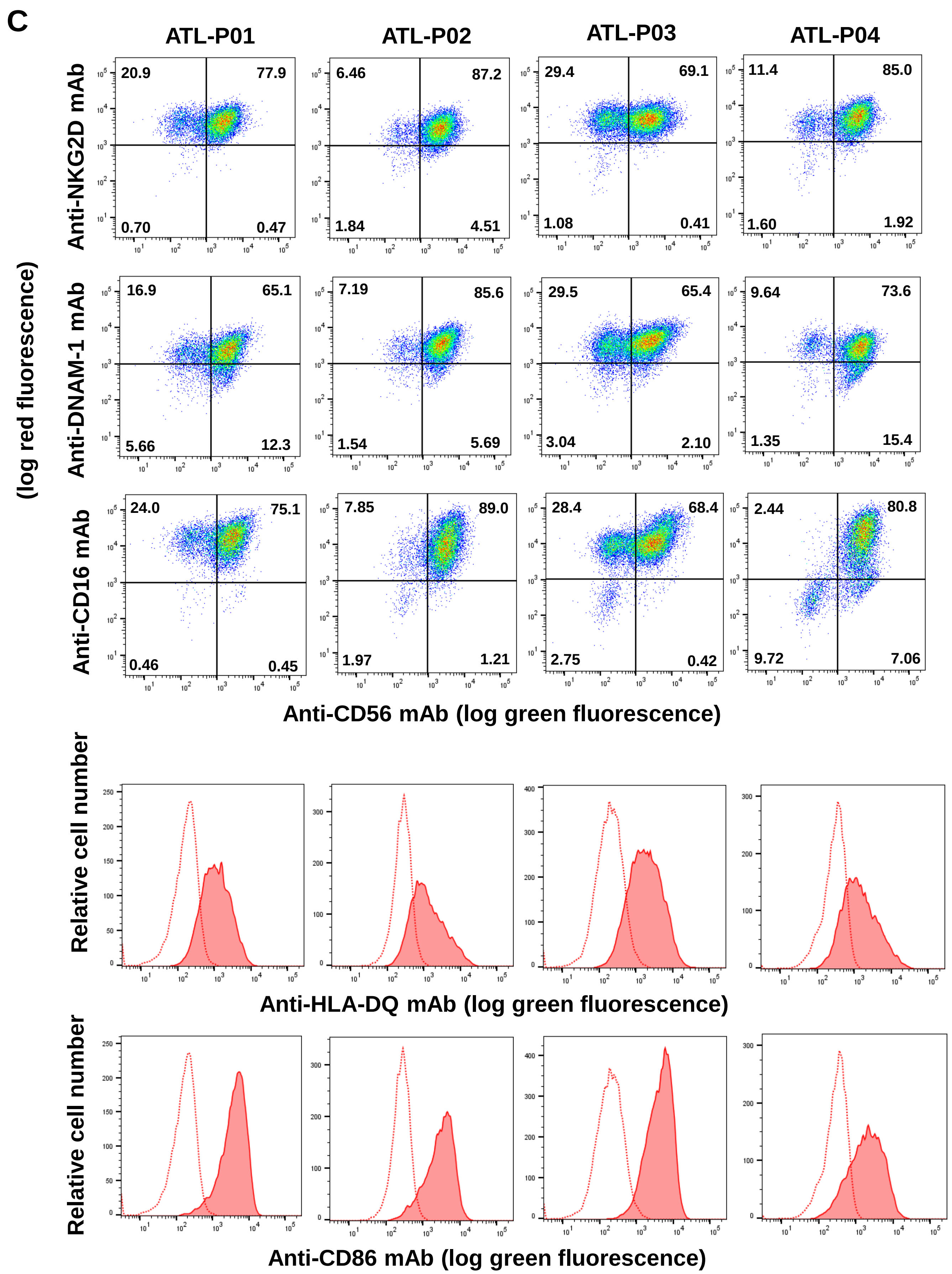
C
ATL-P01
ATL-P02
ATL-P03
ATL-P04
(log red fluorescence)
Anti-NKG2D mAb
20.9
77.9
0.70
0.47
6.46
87.2
1.84
4.51
29.4
69.1
1.08
0.41
11.4
85.0
1.60
1.92
Anti-DNAM-1 mAb
16.9
65.1
5.66
12.3
7.19
85.6
1.54
5.69
29.5
65.4
3.04
2.10
9.64
73.6
1.35
15.4
Anti-CD16 mAb
24.0
75.1
0.46
0.45
7.85
89.0
1.97
1.21
28.4
68.4
2.75
0.42
2.44
80.8
9.72
7.06
Anti-CD56 mAb (log green fluorescence)
Relative cell number
Anti-HLA-DQ mAb (log green fluorescence)
Relative cell number
Anti-CD86 mAb (log green fluorescence)

Supplementary Fig. 5. Expansion with IL-2/IL-18 of NK cells from ATL patients. (A) Flow cytometric analyses of IL-2/IL-18-mediated expansion of NK cells derived from ATL patients. Before and after expansion with IL-2/IL-18, the cells were stained with PE-labeled anti-CD56 mAb and FITC-labeled anti-CD3 mAb and analyzed through a FACS Lyric flow cytometer. **(B) IL-2/IL-18-mediated clustering of NK cells.** After stimulation with IL-2/IL-18, the cell clustering was monitored under a microscope equipped with a CCD camera. **(C) Flow cytometric analysis of cell surface markers on IL-2/IL-18-expanded NK cells.** After expansion with IL-2/IL-18 for 10 days, the cells were stained with PE-labeled anti-NKG2D, DNAM-1, CD16, HLA-DQ, or CD86 mAb and FITC-labeled anti-CD56 mAb and analyzed through a FACS Lyric flow cytometer.

Materials and Methods for Supplementary Fig. 5.
(A) Derivation of NK cells from PBMC obtained from ATL patients: PBMC were purified from ATL patients-derived PBMC as described in Supplementary Fig. 1, from which NK cells were prepared as described in Supplementary Fig. 2.

(B) Flow cytometric analysis: Immunohistochemical staining was performed using FITC-conjugated anti-CD3 mAb (Thermo Fisher Scientific Inc.) and anti-CD56 mAb (BioLegend); and PE-conjugated anti-CD56, anti-NKG2D, anti-DNAM-1, anti-CD16, anti-HLA-DQ, and anti-CD86 mAbs (BioLegend). The stained cells were analyzed using a FACS Lyric flow cytometer (Becton Dickenson) and the cell population was visualized with FlowJo ver. 10.8.1 (FlowJo LLC) as described in Supplementary Fig. 2.

Results for Supplementary Fig. 5. Expansion with IL-2/IL-18 of NK cells from ATL patients.
(A)Flow cytometric analyses of IL-2/IL-18-mediated expansion of NK cells derived from ATL patients. Four representative flow cytometry diagrams out of 28 ATL patients, ATL-P01–04. The median proportion of $CD56^{+}CD3^{-}$ NK cells after $CD3^{+}$ T cell-depletion was 46.6% (range: 0.45%–87.1%). The proportion of NK cells derived from ATL patients before expansion was comparable to that of HDs. When $CD3^{-}$ PBMC fractions derived from 28 ATL patients were stimulated with IL-2/IL-18 for 10 days, the median proportion of NK cells increased to 92.3% (range: 12.1%–98.5%). The median numbers of NK cells (per mL of blood) before and after expansion were 4.08×10^{5} (range: 2.8×10^{4}–3.52×10^{6}) and 4×10^{6} (range: 4×10^{4}– 3.6×10^{7}), respectively. The median expansion rate of NK cells was 11.6-fold (range: 0.1–78.8). Highly purified NK cells were obtained from ATL patients using the IL-2/IL-18 stimulation/expansion system as in the case of HDs. The expansion rate of NK cells from ATL patients was, however, significantly lower than that of HDs ($p = 0.0235$) (Not shown in the figure).

(B) IL-2/IL-18-mediated clustering of NK cells. Microscopic analysis revealed that NK cells derived from ATL patients started to form clusters 4 to 5 days after stimulation with IL-2/IL-18 as in the case of HDs.

(C) Flow cytometric analysis of cell surface markers on IL-2/IL-18-expanded NK cells. On flow cytometric analysis, essentially all the expanded NK cells expressed NKG2D, DNAM-1 and CD16. The median proportions of NKG2D and DNAM-1 in NK cells were 99.01% (range: 91.37%–99.88%) and 93.34% (range: 70.81%–99.19%), respectively. NK cells expressed a high level of CD16; in fact, the median proportion of CD16 in NK cells was 95.08% (range: 70.54%–99.51%). The median proportion of HLA-DQ and CD86 in NK cells was 46.78% (range: 13.57%–81.19%) and 80.38% (range: 52.07%–94.75%), respectively. No significant differences in the expression of NKG2D, DNAM-1, CD16, HLA-DQ, and CD86 were observed between ATL patients and HDs.

Supplementary Note added to Result section 3.5.

Expansion of γδ T cells derived from elderly non-ATL patients. Since most ATL patients are elderly, it is essential to examine the effect of aging on the phenotype and immunological properties of γδ T cells and NK cells to distinguish the effect of the HTLV-1 infection status and age. We obtained peripheral blood samples from 10 elderly non-ATL patients, whose median age was comparable to that of ATL patients.

PMBC derived from 10 elderly non-ATL patients were stimulated/expanded with PTA/IL-2/IL18. The median proportion of γδ T cells in $CD3^+$ lymphocyte fractions before expansion was 0.64% (range: 0.13%–2.52%). None of the elderly non-ATL patients exhibited less than 0.1% of γδ T cells in $CD3^+$ lymphocyte fractions. After expansion of PBMCs with PTA/IL-2/IL-18 for 11 days, the median proportion of γδ T cells in $CD3^+$ lymphocyte fractions increased to 92.90% (range: 72.27%–99.27%). The median numbers of γδ T cells (per mL of blood) before and after expansion were 4×10^3 (range: 4×10^2–3.6×10^4) and 8.5×10^6 (range: 4×10^5–6×10^7), respectively. The median expansion rate of γδ T cells was 2278-fold (range: 180–8089).

On flow cytometric analyses 11 days after stimulation/expansion with PTA/IL-2/IL-18, the median proportions of NKG2D and DNAM-1 in γδ T cells were 97.43% (range: 89.3%–99.89%) and 97.86% (range: 93.5%–99.96%), respectively. γδ T cells expressed CD16 to different degrees; the median proportion of γδ T cells expressing CD16 was 16.27% (range: 1.19%–86.18%). γδ T cells expressed a low level of PD-1 to different degrees, and the median proportion of γδ T cells expressing PD-1 was 15.31% (range: 2.44%–91.2%).

Expansion of NK cells derived from elderly non-ATL patients. $CD3^-$ PBMC derived from elderly non-ATL patients were stimulated with IL-2/IL-18 for 10 days. The median proportion of $CD56^+CD3^-$ NK cell fractions after $CD3^+$ T cell depletion was 40.55% (range: 2.61%–59.8%). After 10 days of incubation, the median proportion of NK cells increased to 93.1% (range: 79.6%–98.5%). The median numbers of NK cells (per mL of blood) before and after expansion were 2.86×10^3 (range: 2.4×10^4–8.48×10^3) and 5×10^6 (range: 6×10^5–1.2×10^7), respectively. The median expansion rate of NK cells was 26.1-fold (range: 1.2–72.5).

On flow cytometric analyses 10 days after IL-2/IL-18 stimulation/expansion, the median proportions of NKG2D and DNAM-1 in γδ T cells were 99.13% (range: 98.35%–99.87%) and 94.34% (range: 85.33%–99.69%), respectively. NK cells expressed a high level of CD16; in fact, the median proportion of NK cells expressing CD16 was 95.52% (range: 85.1%–99.31%). The median proportions of HLA-DQ and CD86 in NK cells were 59.71% (range: 35.29%–86.45%) and 81.55% (range: 46.32%–98.95%), respectively.

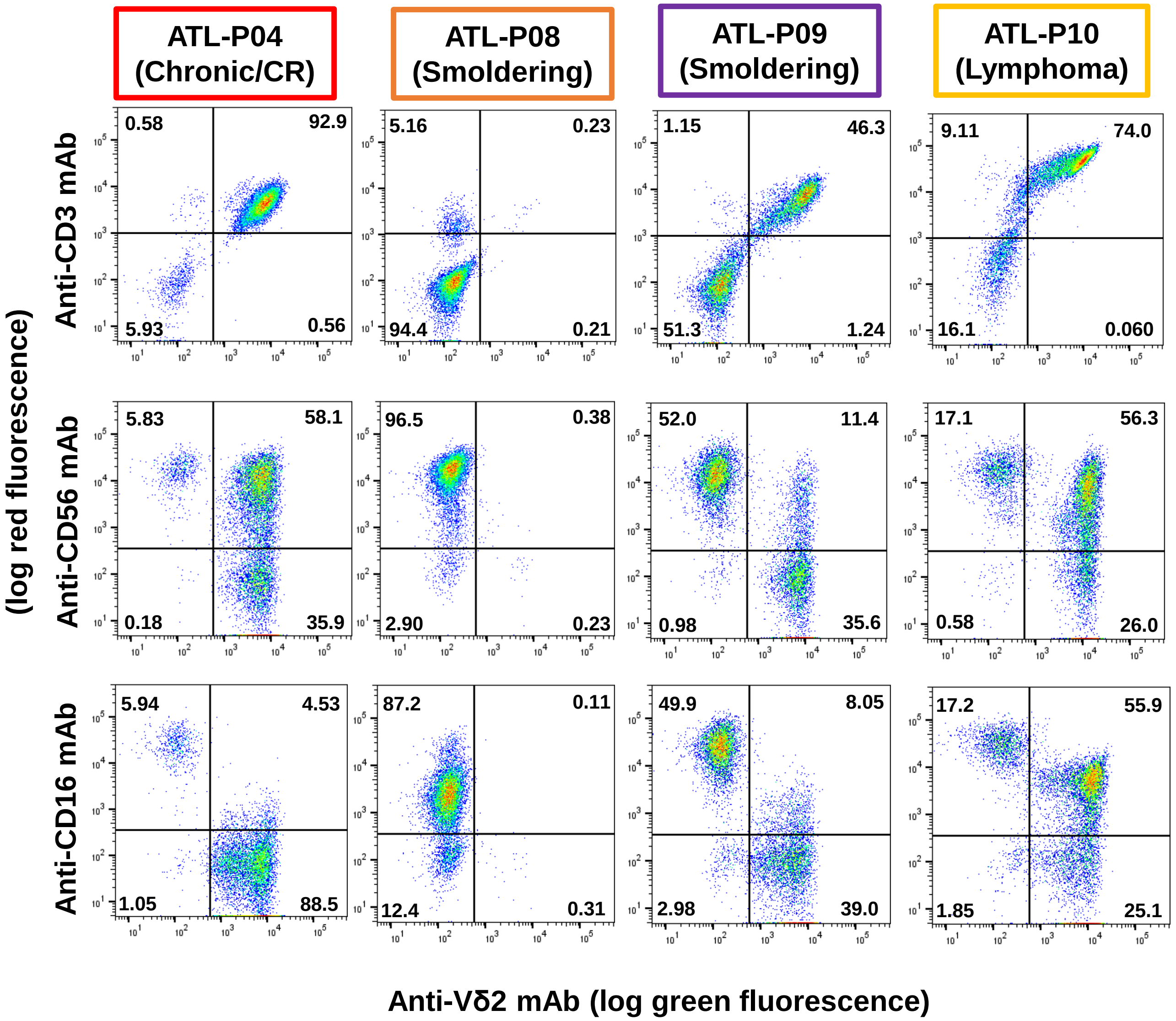

Supplementary Fig. 6. Expansion with PTA/IL-2/IL-18 of γδ T cells and NK cells derived from ATL patients (ATL-P04, 08-14). PBMCs were purified from peripheral blood derived from ATL patients and stimulated/expanded with PTA/IL-2/IL-18, which were stained with PE-conjugated anti-CD3, anti-56 or anti-CD16 mAb and FITC-conjugated anti-Vδ2 mAb, and analyzed through a FACS Lyric flow cytometer.

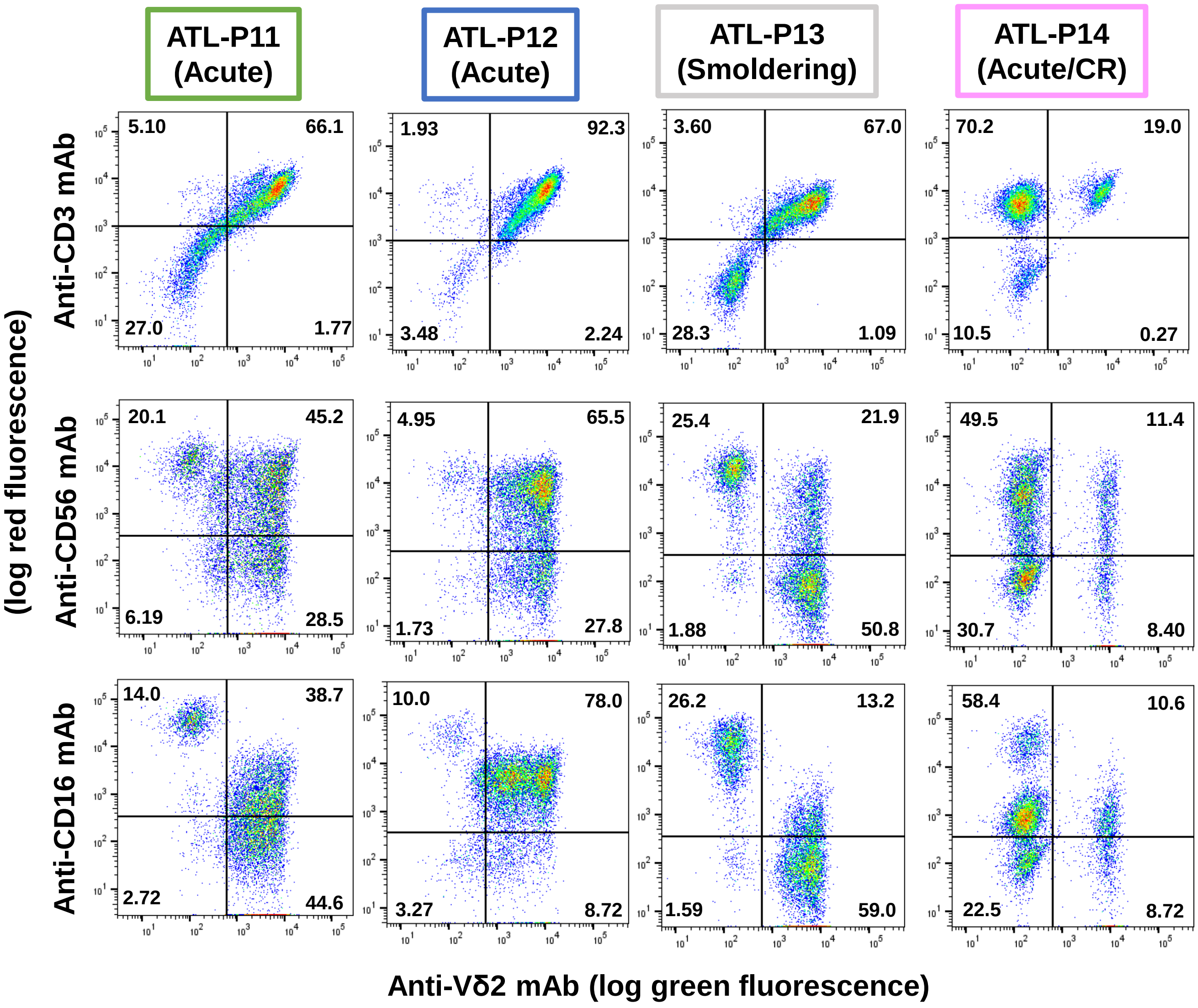

Supplementary Fig. 6. Expansion with PTA/IL-2/IL-18 of γδ T cells and NK cells derived from ATL patients (ATL-P04, 08-14). After stimulation/expansion of PBMCs derived from ATL patients (ATL-P04, 08-14) with PTA/IL-2/IL-18, the cells were stained with PE-conjugated anti-cD3, anti-56 or anti-CD16 mAb and FITC-conjugated anti-Vδ2 mAb, and analyzed through a FACS Lyric flow cytometer.

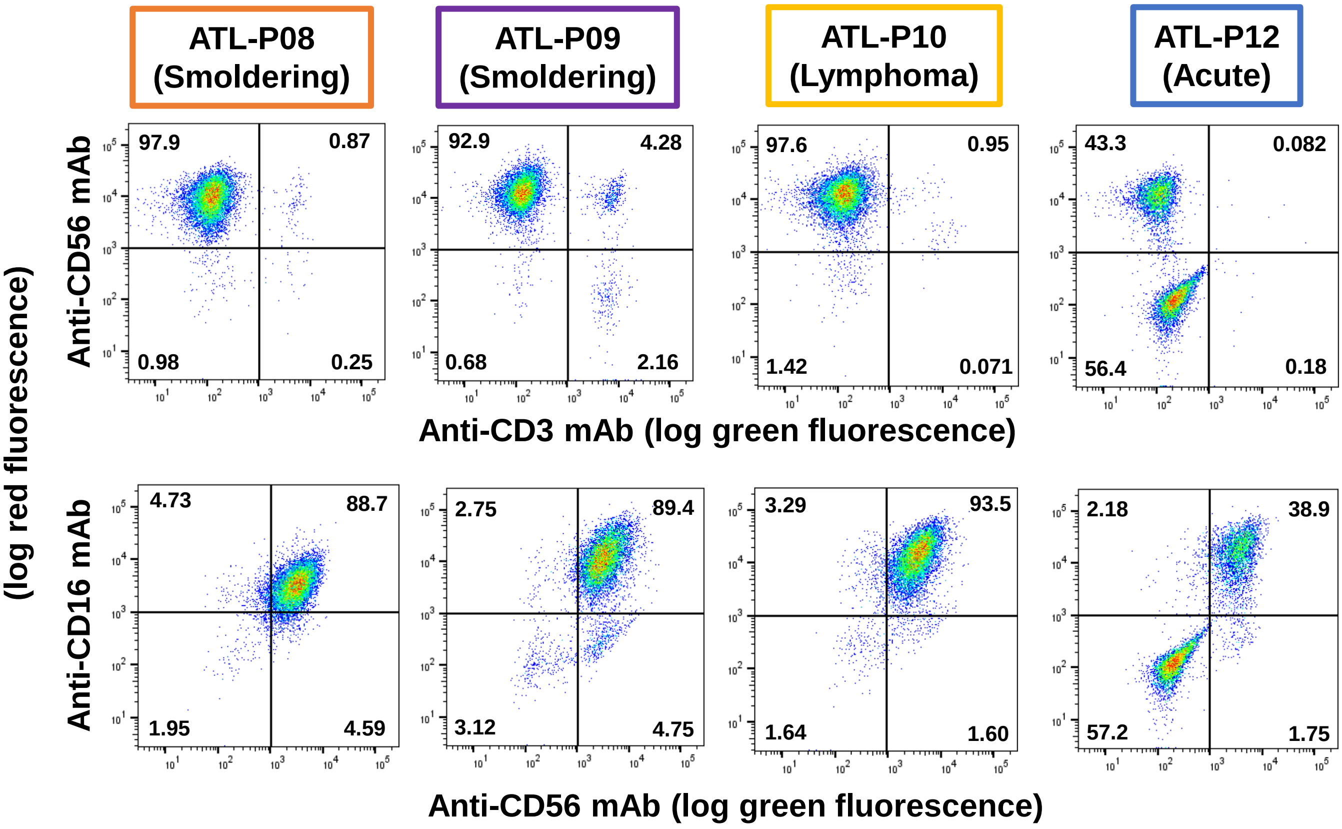

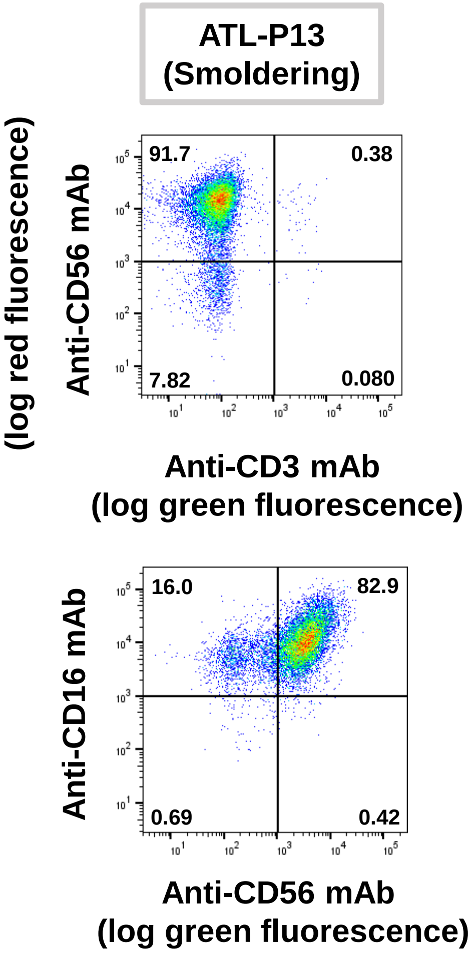

Supplementary Fig. 7. Expansion with IL-2/IL-18 of NK cells derived from ATL patients (ATL-P08-10, 12-13), which were used for the cytotoxicity assay in Fig. 8. PBMCs were purified from peripheral blood derived from ATL patients and stimulated/expanded with IL-2/IL-18, which were stained with PE-conjugated anti-CD56 or anti-CD16 mAb and FITC-conjugated anti-CD3 mAb, and analyzed through a FACS Lyric flow cytometer.

Supplementary Table S1. Summary of flow cytometric analysis, PTA/IL-2-induced expansion of γδ T cells and IL-2/IL-18-induced expansion of NK cells in HDs.

Young, health donors	FACS	PTA/IL-2	PTA/IL-2/IL-18	IL-2/IL-18
HD01	○	○		
HD02	○	○		○
HD03	○	○		
HD04	○	○		
HD05	○			○
HD06	○			○
HD07	○			○
HD08	○	○		
HD09	○	○		
HD10	○	○		
HD11	○			○
HD12	○			○
HD13	○			○
HD14	○	○		○
HD15	○	○		○
HD16	○	○		○

Supplementary Table S2. Summary of the clinical characteristics of 55 ATL patients. ATL, adult T-cell leukemia–lymphoma; mogamulizumab, defucosylated anti-CCR4 monoclonal antibody; PSL, prednisolone; bexarotene, retinoid X receptor (RXR) agonist; lenalidomide, potent inhibitor of TNF-α; sIL-2R, soluble interleukin-2 receptor; LDH, lactate dehydrogenase; Alb, albumin; BUN, blood urea nitrogen; Ca, calcium; corrected Ca = serum Ca + 0.8*(normal albumin - patient albumin); WBCs, white blood cells; Ly, lymphocyte; Ab-Ly, abnormal lymphocyte; IQR, interquartile range.

Characteristic	n = 55
Male sex, n (%)	29 (52.7)
Age at blood sampling, y, median (min, max, IQR)	72 (34, 86, 63.5-78)
Shimoyama classification at diagnosis, n (%)	
Smoldering subtype	13 (23.6)
Favorable chronic subtype	9 (16.4)
Unfavorable chronic subtype	5 (9.1)
Lymphoma subtype	4 (7.3)
Acute subtype	24 (43.6)
Performance status (PS)	1 (0, 4, 0-1)
Previous treatments at the time of blood sampling, n (%)	
Untreated (with no anticancer drugs)	31 (56.4)
Undergoing anticancer drug treatment	10 (18.2)
Post anticancer drug treatment	14 (25.5)
Breakdown of anticancer drug treatment, n (%)	
Only chemotherapy	12 (21.8)
Only mogamulizumab	1 (1.8)
Chemotherapy + mogamulizumab	7 (12.7)
Chemotherapy + lenalidomide	1 (1.8)
Bexarotene	3 (5.5)
Use of PSL or immunosuppressants at the time of blood sampling	
PSL	6 (10.9)
Immunosuppressants	1 (1.8)
Laboratory examinations, median (min, max, IQR)	
sIL-2R (U/ml)	969 (60, 100000, 634.5-4379)
LDH (IU/L)	214 (94, 3882, 72-266)
Alb (g/dL)	4.1 (2.1, 4.7, 3.8-4.3)
BUN (mg/dL)	16 (7, 37, 13-20)
Corrected Ca (mg/dL)	9.5 (8.7, 11, 9.2-9.7)
WBCs ($\times 10^9$/L）	6.6 (2.8, 202.4, 4.825-10.15)
Ly (%)	28 (0, 84, 13-39)
Ab-Ly (%)	3 (0, 96, 0-12)

Supplementary Table S3. The Shimoyama classification at the first diagnosis and the outcome at the time of blood sampling. CR, complete response; PR, partial response.

Shimoyama Classification at Diagnosis	Patients Who Underwent Blood Sampling	Patients who Achieved CR	Patients who Achieved PR
Smoldering subtype (n=13)	3	0	3
Favorable chronic subtype (n=9)	3	2	0
Unfavorable chronic subtype (n=5)	1	0	0
Lymphoma subtype (n=4)	2	2	0
Acute subtype (n=24)	15	9	3

A total of 55 ATL patients (29 males and 26 females) in the Departments of Hematology and Dermatology of Nagasaki University Hospital were enrolled in this study between April 2013 and January 2023. We selected patients with a definitive diagnosis of ATL at the Department of Hematology before blood sampling, using evidence of the monoclonal integration of HTLV-1 proviral DNA and clinical and laboratory findings. Patient information was retrospectively collected, beginning at the time the blood sampling was conducted. Clinical characteristics of 55 ATL patients are summarized in Supplementary Table 2. The median age at the time of blood sampling was 72 years (range: 34 – 86 years). The most common subtype of ATL at first diagnosis, based on the Shimoyama classification [8], was the acute subtype (43.6%). Patients who underwent hematopoietic stem cell transplantation were excluded. The Shimoyama classification at the first diagnosis and the outcome at the time of the blood sampling are summarized in Table 2. Among the ATL patients enrolled, 31 (56.4%) had not received any anticancer drugs, and 24 (43.6%) had a history of anticancer drug use at the time of blood collection. Of these, 6 patients were undergoing chemotherapy treatment and 4 patients were taking lenalidomide or bexarotene internally. At the same time, the following laboratory tests were conducted, all of which were previously established biomarkers for aggressive subtype factors of ATL: levels of serum soluble interleukin-2 receptor (sIL-2R) (U/mL), lactate dehydrogenase (LDH, IU/L), albumin (Alb. g/dL), blood urea nitrogen (BUN, mg/dL), corrected calcium (Ca, mg/dL), white blood cells (WBCs $\times 10^9$/L), lymphocyte (Ly, %), and abnormal lymphocytes (Ab-Ly, %),.

ATL's onset requires a long latency period of approximately 50 – 60 years after infection with HTLV-1 in infants, and ATL, thus, occurs mostly in elderly HTLV-1-infected individuals. Aging is reported to result in the remodeling of T-cell immunity and to be associated with poor clinical outcomes in age-related diseases [61]. In addition, the immune system is also reported to be suppressed in ATL patients [24-26]. It is, therefore, a prerequisite to examine the immunological properties of γδ T cells and NK cells from ATL patients and the effects of aging and immunosuppression status associated with HTLV-1 infections on the effector functions of innate immune cells. To examine the effect of aging on γδ T-cell populations in PBMCs, 10 elderly non-ATL patients (8 males and 2 females) were enrolled in this study; who suffered from epidermal cyst, atopic dermatitis, skin ulcer, alopecia, angioleiomyoma, post-herpes zoster, and prurigo.

Supplementary Table S4. Summary of flow cytometric analysis, PTA/IL-2-induced expansion of γδ T cells, PTA/IL-2/IL-18-induced expansion of γδ T cells and IL-2/IL-18-induced expansion of NK cells in ATL patients.

ATL Patient	FACS	PTA/ IL-2	PTA/ IL-2/IL-18	IL-2/ IL-18	ATL Patient	FACS	PTA/ IL-2	PTA/ IL-2/IL-18	IL-2/ IL-18
ATL-P01	○		○	○	ATL-P29	○		○	○
ATL-P02	○		○	○	ATL-P30	○		○	○
ATL-P03	○		○	○	ATL-P31	○	○	○	
ATL-P04	○		○	○	ATL-P32	○	○	○	
ATL-P05	○		excluded	○	ATL-P33	○	○	○	
ATL-P06	○		○	○	ATL-P34	○	○	○	
ATL-P07	○		○	○	ATL-P35	○	○	○	
ATL-P08	○		○	○	ATL-P36	○	○	○	
ATL-P09	○		○	○	ATL-P37	○	○	○	
ATL-P10	○		○	○	ATL-P38	○	○	○	
ATL-P11	○		○	○	ATL-P39	○	○	○	
ATL-P12	○		○	○	ATL-P40	○	○	○	
ATL-P13	○		○	○	ATL-P41	○	○	○	
ATL-P14	○		○	○	ATL-P42	○	○	○	
ATL-P15	○		○	○	ATL-P43	○	○	○	
ATL-P16	○		excluded	○	ATL-P44	○	○	○	
ATL-P17	○		○	○	ATL-P45	○	○	○	
ATL-P18	○		○	○	ATL-P46	○	○	○	
ATL-P19	○		○	○	ATL-P47	○	○	○	
ATL-P20	○		○	○	ATL-P48	○	○	○	
ATL-P21	○		○	○	ATL-P49	○	○	○	
ATL-P22	○		○	○	ATL-P50	○	○	○	
ATL-P23	○		○	○	ATL-P51	○	○	○	
ATL-P24	○		○	○	ATL-P52	○	○	○	
ATL-P25	○		○	○	ATL-P53	○	○	○	
ATL-P26	○		○	○	ATL-P54	○	○	○	
ATL-P27	○		○	○	ATL-P55	○	○	○	
ATL-P28	○		○	○					

Supplementary Table S5. Summary of flow cytometric analysis, PTA/IL-2/IL-18-induced expansion of γδ T cells and IL-2/IL-18-induced expansion of NK cells in elderly non-ATL patients.

Elderly non-ATL patients	FACS	PTA/IL-2	PTA/IL-2/IL-18	IL-2/IL-18
Elderly-P01	○		○	○
Elderly-P02	○		○	○
Elderly-P03	○		○	○
Elderly-P04	○		○	○
Elderly-P05	○		○	○
Elderly-P06	○		○	○
Elderly-P07	○		○	○
Elderly-P08	○		○	○
Elderly-P09	○		○	○
Elderly-P10	○		○	○
